# Crystal structure of *N*′-[(1*E*)-1-(6-methyl-2,4-dioxo-3,4-di­hydro-2*H*-pyran-3-yl­idene)eth­yl]benzene­sulfono­hydrazide

**DOI:** 10.1107/S1600536814022648

**Published:** 2014-10-24

**Authors:** Jonnie N. Asegbeloyin, Oguejiofo T. Ujam, Chizoba M. Ngige, Valentine I. Onwukeme, Tania Groutso

**Affiliations:** a Department of Pure and Industrial Chemistry, University of Nigeria, Nsukka, Enugu State, Nigeria; b Department of Pure and Industrial Chemistry, Nnamdi Azikiwe University, PMB 5025, Awka, Anambra State, Nigeria; c School of Chemical Sciences, the University of Auckland Private Bag 92019, Auckland 1142, New Zealand

**Keywords:** crystal structure, hydrazone, benzene­sulfono­hydrazide, enamine tautomeric form, hydrogen bonds

## Abstract

In the title compound, C_14_H_14_N_2_O_5_S, the mol­ecule exists in the enamine (C=C—NH) tautomeric form. The hydrazone fragment derived from the 3-acetyl-4-hy­droxy-6-methyl-2*H*-pyran-2-one moiety is approximately planar, with a maximum deviation of 0.1291 (11) Å for the N atom bound to the S atom of the benzensulfono­hydrazide group. The latter adopts a *gauche* conformation relative to the hydrazone N—N bond, with an N—N—S angle of 113.54 (10)°. There is an intra­molecular N—H⋯O=C hydrogen bond that stabilizes the tautomeric form. In the crystal, mol­ecules are linked by N—H⋯O=C hydrogen bonds into chains extending parallel to [100].

## Related literature   

3-Acetyl-4-hy­droxy-6-methyl-2*H*-pyran-2-one and its derivatives have received attention due to their coordination chemistry, pharmaceutical significance and biologically activities (Battaini *et al.*, 2000 ▶; Puerta & Cohen, 2003 ▶; Rao & Narasaiah, 2003 ▶; Zucolotto Chalaça *et al.*, 2002 ▶; Fouad *et al.*, 2010 ▶; Kubaisi & Ismail, 1994 ▶; Rao *et al.*, 1985 ▶; Deshmukh *et al.*, 2010*a* ▶,*b* ▶; Munde *et al.*, 2009 ▶, 2010 ▶; Faidallah *et al.*, 2011 ▶; Jadhav *et al.*, 2010 ▶). 3-Acetyl-4-hy­droxy-6-methyl-2*H*-pyran-2-one is also well-noted for its fungicidal (Rao *et al.*, 1978 ▶), herbicidal and anti­microbial activities (Zucolotto Chalaça *et al.*, 2002 ▶). The title compound is a new hydrazone prepared as part of an on-going research to study the ligating ability and anti­microbial properties of 3-acetyl-4-hy­droxy-6-methyl-2*H*-pyran-2-one hydrazones and their derivatives. For the crystal structure of a related thio­semicarbazone, see: Vrdoljak *et al.* (2008 ▶). For a benzene­sulfono­hydrazide derivative of a similar tautomeric enamine form, see: Ukwueze *et al.* (2014 ▶).
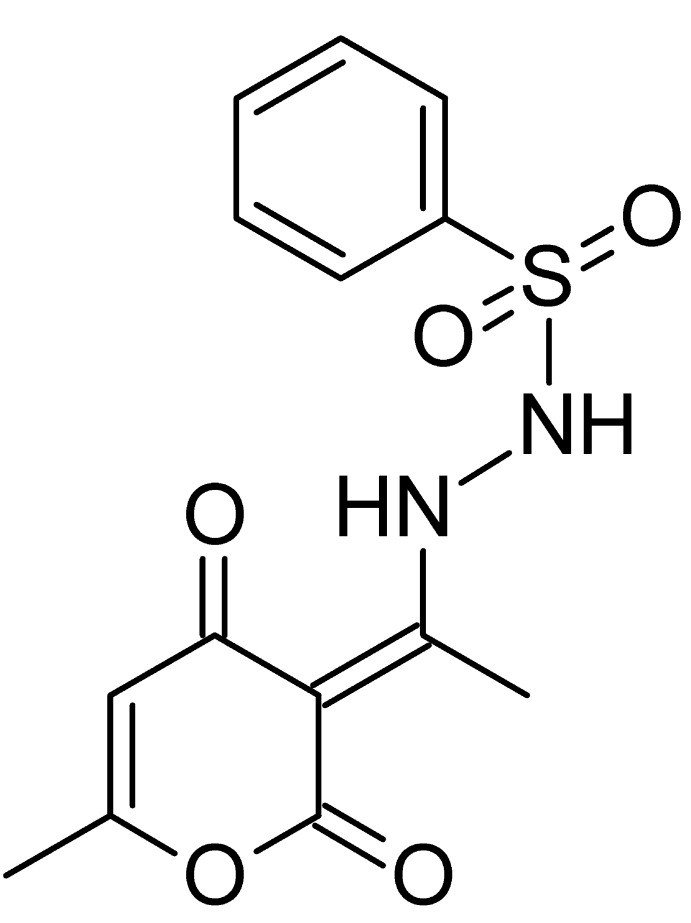


## Experimental   

### Crystal data  

C_14_H_14_N_2_O_5_S
*M* *_r_* = 322.33
Monoclinic, 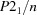

*a* = 7.4797 (4) Å
*b* = 15.2458 (7) Å
*c* = 12.7820 (6) Å
β = 94.282 (3)°
*V* = 1453.51 (12) Å^3^
*Z* = 4
Mo *K*α radiation
μ = 0.25 mm^−1^
*T* = 99 K
0.30 × 0.30 × 0.12 mm

### Data collection  

Bruker APEXII CCD diffractometer
Absorption correction: multi-scan (*SADABS*; Sheldrick, 2003 ▶) *T*_min_ = 0.659, *T*_max_ = 0.746
17170 measured reflections
3484 independent reflections
2817 reflections with *I* > 2σ(*I*)
*R*_int_ = 0.056

### Refinement  

*R*[*F* ^2^ > 2σ(*F* ^2^)] = 0.043
*wR*(*F* ^2^) = 0.126
*S* = 1.05
3484 reflections
209 parameters
H atoms treated by a mixture of independent and constrained refinement
Δρ_max_ = 0.35 e Å^−3^
Δρ_min_ = −0.42 e Å^−3^

### 

Data collection: *APEX2* (Bruker, 2004 ▶); cell refinement: *SAINT* (Bruker, 2004 ▶); data reduction: *SAINT*; program(s) used to solve structure: *SHELXS97* (Sheldrick, 2008 ▶); program(s) used to refine structure: *SHELXL2013* (Sheldrick, 2008 ▶); molecular graphics: *ORTEP-3 for Windows* (Farrugia, 2012 ▶) and *Mercury* (Macrae *et al.*, 2006 ▶); software used to prepare material for publication: *WinGX* (Farrugia, 2012 ▶).

## Supplementary Material

Crystal structure: contains datablock(s) I. DOI: 10.1107/S1600536814022648/wm5072sup1.cif

Structure factors: contains datablock(s) I. DOI: 10.1107/S1600536814022648/wm5072Isup2.hkl

Supporting information file. DOI: 10.1107/S1600536814022648/wm5072Isup3.mol

Supporting information file. DOI: 10.1107/S1600536814022648/wm5072Isup4.cml

**Fig. 1.**
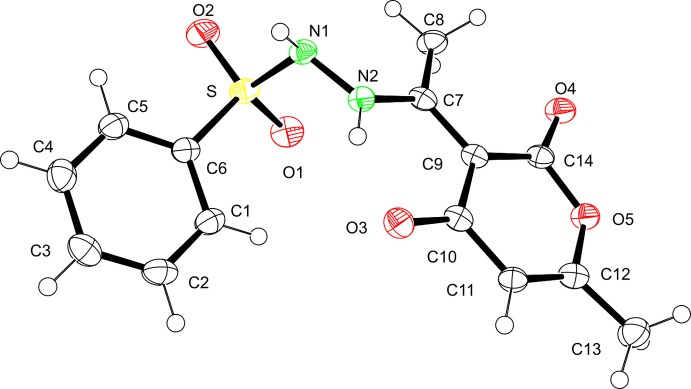
The molecular structure and atom numbering of the title compound with displacement ellipsoids drawn at the 50% probability level for non-H atoms.

. DOI: 10.1107/S1600536814022648/wm5072fig1.tif
The mol­ecular structure and atom numbering of the title compound with displacement ellipsoids drawn at the 50% probability level for non-H atoms.

**Fig. 2.**
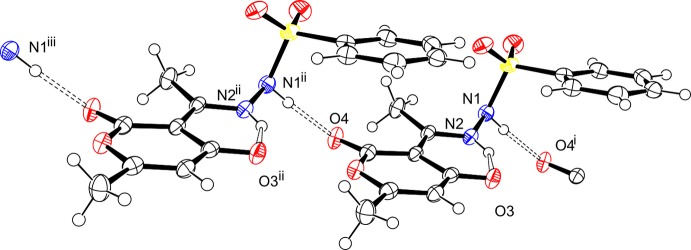
Intramolecular N2–Hn2···O3 and intermolecular N1–Hn1···O4 (dotted lines) hydrogen bonding interactions in the title compound. [Symmetry codes: i) 1+x, y, z; ii) -1+x, y, z; iii) -2+x, y, z.]

. DOI: 10.1107/S1600536814022648/wm5072fig2.tif
Intra­molecular N2–Hn2⋯O3 and inter­molecular N1–Hn1⋯O4 (dotted lines) hydrogen bonding inter­actions in the title compound. [Symmetry codes: i) 1+x, y, z; ii) −1+x, y, z; iii) −2+x, y, z.]

**Fig. 3.**
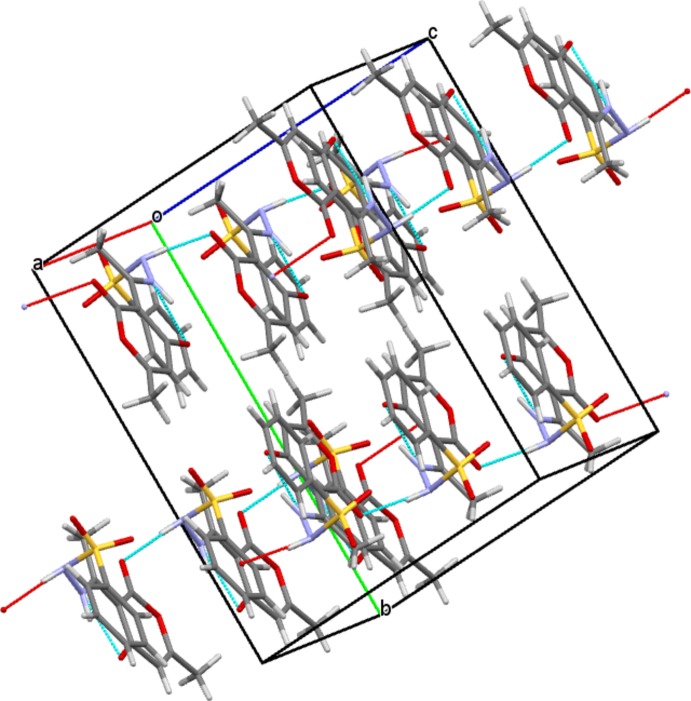
The packing diagram of the title compound showing intra- and intermolecular N—H···O═C hydrogen bonds as dotted lines.

. DOI: 10.1107/S1600536814022648/wm5072fig3.tif
The packing diagram of the title compound showing intra- and inter­molecular N—H⋯O=C hydrogen bonds as dotted lines.

CCDC reference: 1029366

Additional supporting information:  crystallographic information; 3D view; checkCIF report

## Figures and Tables

**Table 1:** Hydrogen-bond geometry (, )

*D*H*A*	*D*H	H*A*	*D* *A*	*D*H*A*
N1H*N*1O4^i^	0.89(2)	1.89(2)	2.7837(19)	175(2)
N2H*N*2O3	0.90(2)	1.74(2)	2.5194(18)	144(2)

Symmetry code: (i) 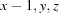.

## supplementary crystallographic information

### S1. Synthesis and Crystallisation

A solution of 3-acetyl-4-hy­droxy-6-methyl-(2H)-pyran-2-one [168 mg, 1 mmol] in methanol (10 ml) was mixed with a solution of benzene­sulfono­hydrazide [172 mg, 1 mmol] in methanol (10 ml). The mixture was refluxed for 3 h, and the resulting solution cooled to obtain a white precipitate. The product was filtered, dried and recrystallized from methanol. Crystals suitable for X-ray crystallographic analysis were obtained by slow evaporation of a methano­lic solution at room temperature for 48 h.

### S2. Refinement

Crystal data, data collection and structure refinement details are summarized in Table 2. Hydrogen atoms were placed in calculated positions with C—H = 0.95 - 0.98 Å and refined using a riding model with displacement parameters *U*_iso_(H) = 1.2 *U*_eq_(C) for aromatic and methyl­ene groups and *U*_iso_(H) = 1.5 *U*_eq_(C) for methyl groups. The two N—H hydrogen atoms were located in a difference map and were refined freely.

### Crystal data

**Table:**

C_14_H_14_N_2_O_5_S	*F*(000) = 672
*M**_r_* = 322.33	*D*_x_ = 1.473 Mg m^−^^3^
Monoclinic, *P*2_1_/*n*	Melting point: 473 K
Hall symbol: -P 2yn	Mo *K*α radiation, λ = 0.71073 Å
*a* = 7.4797 (4) Å	Cell parameters from 5140 reflections
*b* = 15.2458 (7) Å	θ = 2.7–28.0°
*c* = 12.7820 (6) Å	µ = 0.25 mm^−^^1^
β = 94.282 (3)°	*T* = 99 K
*V* = 1453.51 (12) Å^3^	Needle, colourless
*Z* = 4	0.30 × 0.30 × 0.12 mm

### Data collection

**Table:**

Bruker APEXII CCD diffractometer	3484 independent reflections
Radiation source: fine-focus sealed tube	2817 reflections with *I* > 2σ(*I*)
Graphite monochromator	*R*_int_ = 0.056
φ and ω scans	θ_max_ = 28.0°, θ_min_ = 2.1°
Absorption correction: multi-scan (*SADABS*; Sheldrick, 2003)	*h* = −9→9
*T*_min_ = 0.659, *T*_max_ = 0.746	*k* = −20→19
17170 measured reflections	*l* = −16→16

### Refinement

**Table:**

Refinement on *F*^2^	0 restraints
Least-squares matrix: full	Hydrogen site location: mixed
*R*[*F*^2^ > 2σ(*F*^2^)] = 0.043	H atoms treated by a mixture of independent and constrained refinement
*wR*(*F*^2^) = 0.126	*w* = 1/[σ^2^(*F*_o_^2^) + (0.0681*P*)^2^ + 0.4046*P*] where *P* = (*F*_o_^2^ + 2*F*_c_^2^)/3
*S* = 1.05	(Δ/σ)_max_ < 0.001
3484 reflections	Δρ_max_ = 0.35 e Å^−^^3^
209 parameters	Δρ_min_ = −0.42 e Å^−^^3^

### Special details

**Table:**

Geometry. All e.s.d.'s (except the e.s.d. in the dihedral angle between two l.s. planes) are estimated using the full covariance matrix. The cell e.s.d.'s are taken into account individually in the estimation of e.s.d.'s in distances, angles and torsion angles; correlations between e.s.d.'s in cell parameters are only used when they are defined by crystal symmetry. An approximate (isotropic) treatment of cell e.s.d.'s is used for estimating e.s.d.'s involving l.s. planes.

### Fractional atomic coordinates and isotropic or equivalent isotropic displacement parameters (Å^2^)

**Table:**

	*x*	*y*	*z*	*U*_iso_*/*U*_eq_	
C1	−0.1162 (3)	0.17867 (12)	−0.16895 (13)	0.0281 (4)	
H1	0.0092	0.1698	−0.1554	0.034*	
C2	−0.2300 (3)	0.10824 (12)	−0.19238 (14)	0.0326 (4)	
H2	−0.1824	0.0505	−0.1945	0.039*	
C3	−0.4125 (3)	0.12162 (13)	−0.21263 (13)	0.0321 (4)	
H3	−0.4890	0.0732	−0.2298	0.038*	
C4	−0.4844 (3)	0.20534 (13)	−0.20807 (13)	0.0322 (4)	
H4	−0.6099	0.2139	−0.2211	0.039*	
C5	−0.3730 (3)	0.27633 (12)	−0.18449 (12)	0.0280 (4)	
H5	−0.4214	0.3338	−0.1812	0.034*	
C6	−0.1896 (2)	0.26269 (11)	−0.16563 (12)	0.0237 (4)	
C7	0.2970 (2)	0.31837 (11)	0.04228 (12)	0.0216 (3)	
C8	0.3518 (2)	0.41187 (11)	0.02935 (15)	0.0289 (4)	
H8A	0.2446	0.4487	0.0183	0.043*	
H8B	0.4239	0.4169	−0.0313	0.043*	
H8C	0.4228	0.4313	0.0926	0.043*	
C9	0.4199 (2)	0.24839 (10)	0.06583 (11)	0.0206 (3)	
C10	0.3574 (2)	0.15821 (10)	0.06591 (12)	0.0227 (3)	
C11	0.4934 (2)	0.09113 (11)	0.08080 (12)	0.0231 (3)	
H11	0.4581	0.0313	0.0802	0.028*	
C12	0.6670 (2)	0.11099 (11)	0.09535 (12)	0.0256 (4)	
C13	0.8158 (2)	0.04793 (13)	0.11667 (16)	0.0351 (4)	
H13A	0.8639	0.0538	0.1898	0.053*	
H13B	0.9108	0.0602	0.0699	0.053*	
H13C	0.7713	−0.0119	0.1045	0.053*	
C14	0.6075 (2)	0.26625 (11)	0.08402 (12)	0.0231 (3)	
N1	−0.0028 (2)	0.35893 (9)	−0.00836 (11)	0.0236 (3)	
N2	0.12527 (19)	0.29758 (9)	0.03004 (10)	0.0223 (3)	
O1	0.12058 (18)	0.33764 (9)	−0.18100 (10)	0.0336 (3)	
O2	−0.14865 (19)	0.43127 (8)	−0.16350 (10)	0.0361 (3)	
O3	0.19542 (17)	0.13680 (8)	0.05307 (10)	0.0289 (3)	
O4	0.68037 (16)	0.33852 (8)	0.09030 (10)	0.0293 (3)	
O5	0.72498 (16)	0.19616 (8)	0.09491 (9)	0.0260 (3)	
S	−0.04830 (6)	0.35372 (3)	−0.13822 (3)	0.02574 (15)	
HN1	−0.102 (3)	0.3549 (13)	0.0260 (16)	0.033 (5)*	
HN2	0.100 (3)	0.2408 (16)	0.0391 (17)	0.046 (6)*	

### Atomic displacement parameters (Å^2^)

**Table:**

	*U*^11^	*U*^22^	*U*^33^	*U*^12^	*U*^13^	*U*^23^
C1	0.0314 (10)	0.0252 (8)	0.0276 (8)	0.0063 (7)	0.0026 (7)	0.0010 (6)
C2	0.0455 (11)	0.0217 (8)	0.0306 (9)	0.0031 (8)	0.0026 (8)	−0.0002 (7)
C3	0.0413 (11)	0.0308 (9)	0.0238 (8)	−0.0067 (8)	0.0004 (8)	0.0004 (7)
C4	0.0305 (10)	0.0400 (10)	0.0255 (8)	0.0011 (8)	−0.0013 (7)	−0.0011 (7)
C5	0.0327 (10)	0.0274 (9)	0.0237 (8)	0.0071 (8)	0.0011 (7)	−0.0002 (6)
C6	0.0305 (9)	0.0222 (8)	0.0185 (7)	0.0029 (7)	0.0023 (7)	0.0014 (6)
C7	0.0237 (8)	0.0223 (8)	0.0198 (7)	−0.0050 (6)	0.0076 (6)	−0.0039 (6)
C8	0.0258 (9)	0.0192 (8)	0.0423 (10)	−0.0029 (7)	0.0068 (8)	−0.0026 (7)
C9	0.0222 (8)	0.0201 (8)	0.0200 (7)	−0.0036 (6)	0.0059 (6)	−0.0018 (6)
C10	0.0267 (9)	0.0221 (8)	0.0196 (7)	−0.0037 (7)	0.0051 (6)	−0.0011 (6)
C11	0.0256 (9)	0.0186 (7)	0.0255 (7)	−0.0025 (6)	0.0044 (7)	0.0004 (6)
C12	0.0307 (9)	0.0226 (8)	0.0242 (7)	−0.0012 (7)	0.0061 (7)	−0.0005 (6)
C13	0.0269 (10)	0.0306 (10)	0.0476 (11)	0.0019 (8)	0.0015 (8)	−0.0021 (8)
C14	0.0246 (9)	0.0223 (8)	0.0231 (7)	−0.0015 (7)	0.0061 (6)	−0.0035 (6)
N1	0.0219 (7)	0.0230 (7)	0.0265 (7)	0.0009 (6)	0.0059 (6)	−0.0017 (5)
N2	0.0213 (7)	0.0210 (7)	0.0251 (6)	−0.0014 (6)	0.0051 (5)	0.0000 (5)
O1	0.0352 (7)	0.0369 (7)	0.0303 (6)	−0.0015 (6)	0.0129 (6)	0.0034 (5)
O2	0.0450 (8)	0.0206 (6)	0.0422 (7)	0.0044 (6)	−0.0010 (6)	0.0050 (5)
O3	0.0245 (6)	0.0243 (6)	0.0380 (7)	−0.0073 (5)	0.0031 (5)	0.0003 (5)
O4	0.0221 (6)	0.0243 (6)	0.0421 (7)	−0.0050 (5)	0.0072 (5)	−0.0061 (5)
O5	0.0227 (6)	0.0239 (6)	0.0318 (6)	−0.0020 (5)	0.0051 (5)	−0.0020 (5)
S	0.0308 (3)	0.0203 (2)	0.0266 (2)	0.00152 (17)	0.00559 (18)	0.00282 (14)

### Geometric parameters (Å, º)

**Table:**

C1—C2	1.389 (3)	C9—C10	1.452 (2)
C1—C6	1.396 (2)	C10—O3	1.254 (2)
C1—H1	0.9500	C10—C11	1.445 (2)
C2—C3	1.386 (3)	C11—C12	1.332 (2)
C2—H2	0.9500	C11—H11	0.9500
C3—C4	1.388 (3)	C12—O5	1.369 (2)
C3—H3	0.9500	C12—C13	1.481 (3)
C4—C5	1.386 (3)	C13—H13A	0.9800
C4—H4	0.9500	C13—H13B	0.9800
C5—C6	1.391 (3)	C13—H13C	0.9800
C5—H5	0.9500	C14—O4	1.229 (2)
C6—S	1.7635 (18)	C14—O5	1.384 (2)
C7—N2	1.321 (2)	N1—N2	1.401 (2)
C7—C9	1.426 (2)	N1—S	1.6712 (15)
C7—C8	1.496 (2)	N1—HN1	0.89 (2)
C8—H8A	0.9800	N2—HN2	0.90 (2)
C8—H8B	0.9800	O1—S	1.4345 (13)
C8—H8C	0.9800	O2—S	1.4246 (13)
C9—C14	1.431 (2)		
			
C2—C1—C6	118.68 (17)	O3—C10—C9	123.66 (15)
C2—C1—H1	120.7	C11—C10—C9	116.55 (15)
C6—C1—H1	120.7	C12—C11—C10	121.76 (15)
C3—C2—C1	120.40 (17)	C12—C11—H11	119.1
C3—C2—H2	119.8	C10—C11—H11	119.1
C1—C2—H2	119.8	C11—C12—O5	121.38 (16)
C2—C3—C4	120.47 (18)	C11—C12—C13	126.11 (16)
C2—C3—H3	119.8	O5—C12—C13	112.47 (15)
C4—C3—H3	119.8	C12—C13—H13A	109.5
C5—C4—C3	119.92 (18)	C12—C13—H13B	109.5
C5—C4—H4	120.0	H13A—C13—H13B	109.5
C3—C4—H4	120.0	C12—C13—H13C	109.5
C4—C5—C6	119.38 (16)	H13A—C13—H13C	109.5
C4—C5—H5	120.3	H13B—C13—H13C	109.5
C6—C5—H5	120.3	O4—C14—O5	114.24 (15)
C5—C6—C1	121.14 (17)	O4—C14—C9	127.28 (16)
C5—C6—S	119.04 (13)	O5—C14—C9	118.48 (14)
C1—C6—S	119.81 (14)	N2—N1—S	113.54 (10)
N2—C7—C9	116.83 (14)	N2—N1—HN1	110.8 (13)
N2—C7—C8	119.20 (16)	S—N1—HN1	111.7 (14)
C9—C7—C8	123.96 (15)	C7—N2—N1	120.97 (14)
C7—C8—H8A	109.5	C7—N2—HN2	115.3 (16)
C7—C8—H8B	109.5	N1—N2—HN2	123.2 (16)
H8A—C8—H8B	109.5	C12—O5—C14	122.25 (13)
C7—C8—H8C	109.5	O2—S—O1	121.29 (8)
H8A—C8—H8C	109.5	O2—S—N1	104.51 (8)
H8B—C8—H8C	109.5	O1—S—N1	105.48 (8)
C7—C9—C14	120.05 (14)	O2—S—C6	108.08 (8)
C7—C9—C10	120.42 (15)	O1—S—C6	108.75 (8)
C14—C9—C10	119.45 (15)	N1—S—C6	108.02 (7)
O3—C10—C11	119.79 (14)		

### Hydrogen-bond geometry (Å, º)

**Table:**

*D*—H···*A*	*D*—H	H···*A*	*D*···*A*	*D*—H···*A*
N1—H*N*1···O4^i^	0.89 (2)	1.89 (2)	2.7837 (19)	175 (2)
N2—H*N*2···O3	0.90 (2)	1.74 (2)	2.5194 (18)	144 (2)

Symmetry code: (i) *x*−1, *y*, *z*.
