# Supplementary figures and images for: Crystal structure of N′-[(1E)-1-(6-methyl-2,4-dioxo-3,4-di­hydro-2H-pyran-3-yl­idene)eth­yl]benzene­sulfono­hydrazide
Source: Acta Crystallogr Sect E Struct Rep Online. 2014 Oct 24;70(Pt 11):o1179–80. doi: 10.1107/S1600536814022648 (PMC4257349; doi:10.1107/S1600536814022648)

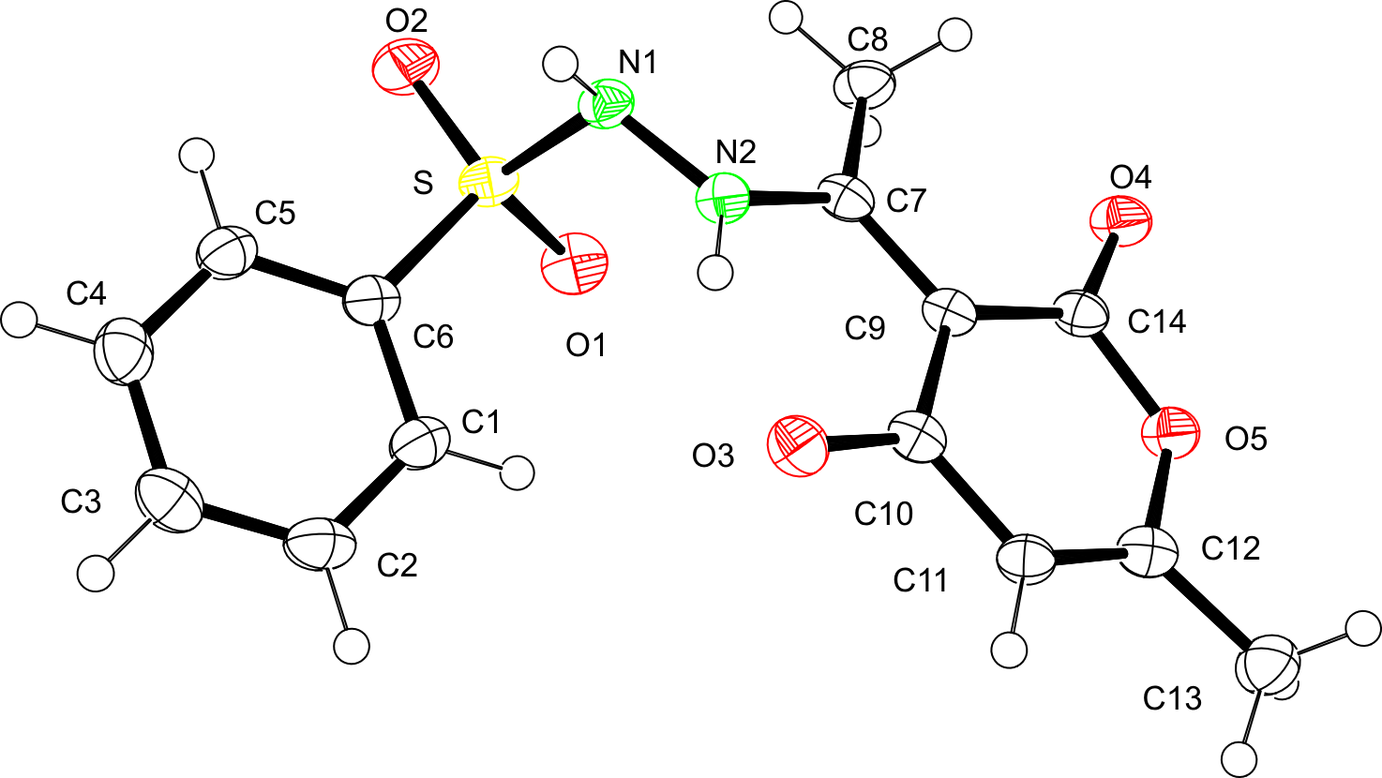

Supplement: Supplementary file 5 [file e-70-o1179-fig1.tif]

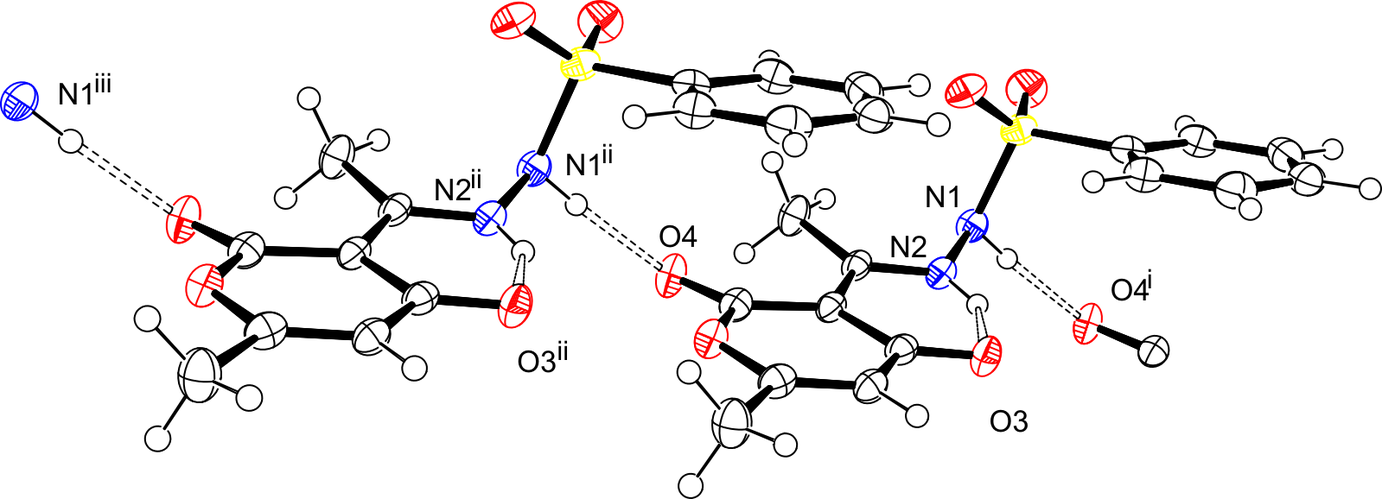

Supplement: Supplementary file 6 [file e-70-o1179-fig2.tif]

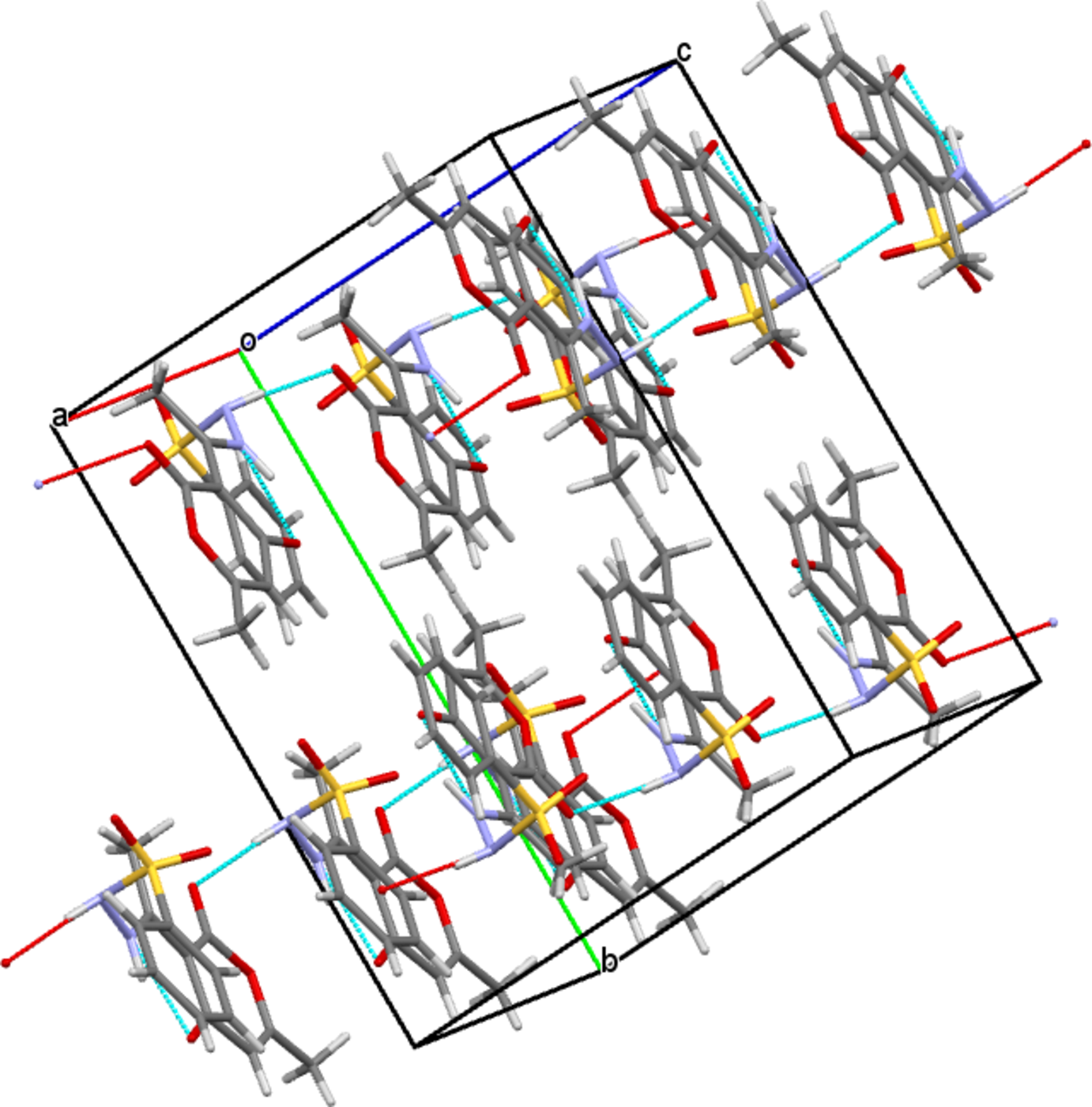

Supplement: Supplementary file 7 [file e-70-o1179-fig3.tif]
